# Astragaloside IV inhibits cell viability and glycolysis of hepatocellular carcinoma by regulating KAT2A-mediated succinylation of PGAM1

**DOI:** 10.1186/s12885-024-12438-9

**Published:** 2024-06-04

**Authors:** Yuanzhang Zhu, Fei Lu

**Affiliations:** Department of Pharmacy, Ruijin Hospital, Shanghai Jiao Tong University School of Medicine, 197 Ruijin Second Road, Huangpu District, Shanghai, 200020 China

**Keywords:** Astragaloside IV, Hepatocellular carcinoma, KAT2A, Cell viability, Glycolysis, PGAM1

## Abstract

**Background:**

Astragaloside IV (AS-IV) is one of the basic components of *Astragali radix*, that has been shown to have preventive effects against various diseases, including cancers. This study aimed to explore the role of AS-IV in hepatocellular carcinoma (HCC) and its underlying mechanism.

**Methods:**

The cell viability, glucose consumption, lactate production, and extracellular acidification rate (ECAR) in SNU-182 and Huh7 cell lines were detected by specific commercial kits. Western blot was performed to analyze the succinylation level in SNU-182 and Huh7 cell lines. The interaction between lysine acetyltransferase (KAT) 2 A and phosphoglycerate mutase 1 (PGAM1) was evaluated by co-immunoprecipitation and immunofluorescence assays. The role of KAT2A in vivo was explored using a xenografted tumor model.

**Results:**

The results indicated that AS-IV treatment downregulated the protein levels of succinylation and KAT2A in SNU-182 and Huh7 cell lines. The cell viability, glucose consumption, lactate production, ECAR, and succinylation levels were decreased in AS-IV-treated SNU-182 and Huh7 cell lines, and the results were reversed after KAT2A overexpression. KAT2A interacted with PGAM1 to promote the succinylation of PGAM1 at K161 site. KAT2A overexpression promoted the viability and glycolysis of SNU-182 and Huh7 cell lines, which were partly blocked following PGAM1 inhibition. In tumor-bearing mice, AS-IV suppressed tumor growth though inhibiting KAT2A-mediated succinylation of PGAM1.

**Conclusion:**

AS-IV inhibited cell viability and glycolysis in HCC by regulating KAT2A-mediated succinylation of PGAM1, suggesting that AS-IV might be a potential and suitable therapeutic agent for treating HCC.

**Supplementary Information:**

The online version contains supplementary material available at 10.1186/s12885-024-12438-9.

## Introduction

Hepatocellular carcinoma (HCC) is a globally threatening cancer worldwide with increasing incidence and mortality, typically developing after liver cirrhosis [1]. HCC is prone to recurrence and metastasis with a 5-year survival rate of 18%, which is much lower than other common cancers [2]. Risk factors for HCC include metabolic liver disorders, alcohol addiction, hereditary hepatitis, and exposure to dietary toxins. Statistical estimations revealed that the global mortality from HCC is expected to reach one million annually worldwide by 2030 [3]. Early HCC can be managed with local ablation, surgical resection, or liver transplantation; but unfortunately, most HCC has been found in the middle or later stages because the liver is not sensitive to pain. Treatment options for patients with advanced HCC are limited in terms of availability and effectiveness [4]. Thus, investigating and exploring novel treatment strategies for HCC is imperative.

Traditional Chinese medicine (TCM) has been recognized worldwide due to its natural origin, low toxicity, limited side effects, and multiple curative effects [5]. Astragaloside IV (AS-IV, 3-O-β-D-xylopyranosyl-6-O-β-D-glucopyranosyl cycloastragenol) is a natural saponin extracted from *Astragali radix* [6], with numerous reported protective therapeutic effects in heart failure [7], diabetic nephropathy [8], neurological disorders [9], and cancers [6, 10]. The role of AS-IV in liver-related diseases has been described previously. For example, AS-IV suppresses oxidative stress by inhibiting the activation of the nuclear factor-κB signal pathway, thereby improving rat liver function and alleviating alcoholic fatty liver disease [11]. AS-IV has also been found to regulate inflammation to protect against lipopolysaccharide-induced liver injury [12], and possesses excellent potential in suppressing HCC progression and metastasis [13, 14].

In recent years, succinylation modification has become a hotspot in post-translational modification research, which regulates the structure of proteins by transferring succinyl groups (-CO-CH_2_-CH_2_-CO_2_H) to residues of target proteins [15]. Succinylation is involved in various physiological processes including mitochondrial metabolism, tricarboxylic acid cycle, and gene regulation [16]. Dysregulation of succinylation is associated with the malignant progression of multiple diseases, including cardiovascular disease [17], metabolic disease [18], and cancers [19]. Carnitine palmitoyltransferase 1 A (CPT1A) succinylates lactate dehydrogenase A, thus promoting the invasion and proliferation of gastric cancer [20]. Down-regulation of sirtuin (SIRT) 5, a desuccinylation enzyme, is associated with the increased succinylation and DNA oxidative damage in HCC [21]. Lysine acetyltransferase (KAT) 2 A, acts as a succinyltransferase, and can be coupled with the metabolic enzyme complex α-ketoglutarate dehydrogenase, affecting the proliferation and formation of tumors [22]. However, the succinylation in HCC has been scarcely studied.

Glycolysis is the common stage through which all organisms undergo glucose catabolism, and impaired glycolysis has been reported to have a a pathological effect on the onset and progression of various diseases, including tumors [23–25]. Cancer cell metabolic reprogramming shows an increase in glycolytic dependence to meet the anabolic needs of cancer cell proliferation [26]. Glycolysis affects tumor proliferation, invasion, microenvironment, and chemotherapy resistance [27]. Previous studies have observed elevated glycolytic activity and lactic acid level in patients with nonalcoholic fatty liver disease and nonalcoholic steatohepatitis [28, 29]. Nevertheless, the effect of glycolysis in HCC remains unknown.

This study aimed to investigate the effects of AS-IV on cell viability and glycolysis in HCC and further explored the underlying mechanism, which might provide a molecular basis for potential targeted HCC therapies.

## Methods and materials

### Cell culture

The SNU-182 and Huh7 cell lines were purchased from Suncell Biotechnology Co., LTD (Wuhan, China). The cells were cultured in Dulbecco’s modified Eagle’s medium (DMEM, Biowit Technology Co., LTD, Shenzhen, China) supplemented with 10% fetal bovine serum (FBS, Thermo Fisher, USA), 100 U/mL penicillin and 100 mg/mL streptomycin (Thermo Fisher). Primary human hepatocytes (PHHs) were obtained from ZEPING Bioscience&Technology Co., LTD (Beijing, China) and cultured in hepatocytes plating medium (ZEPING). The incubated environmental requirement of all cells was 37 °C with 5% CO_2_ in a humidified incubator.

### AS-IV treatment

**Fig. 1:**
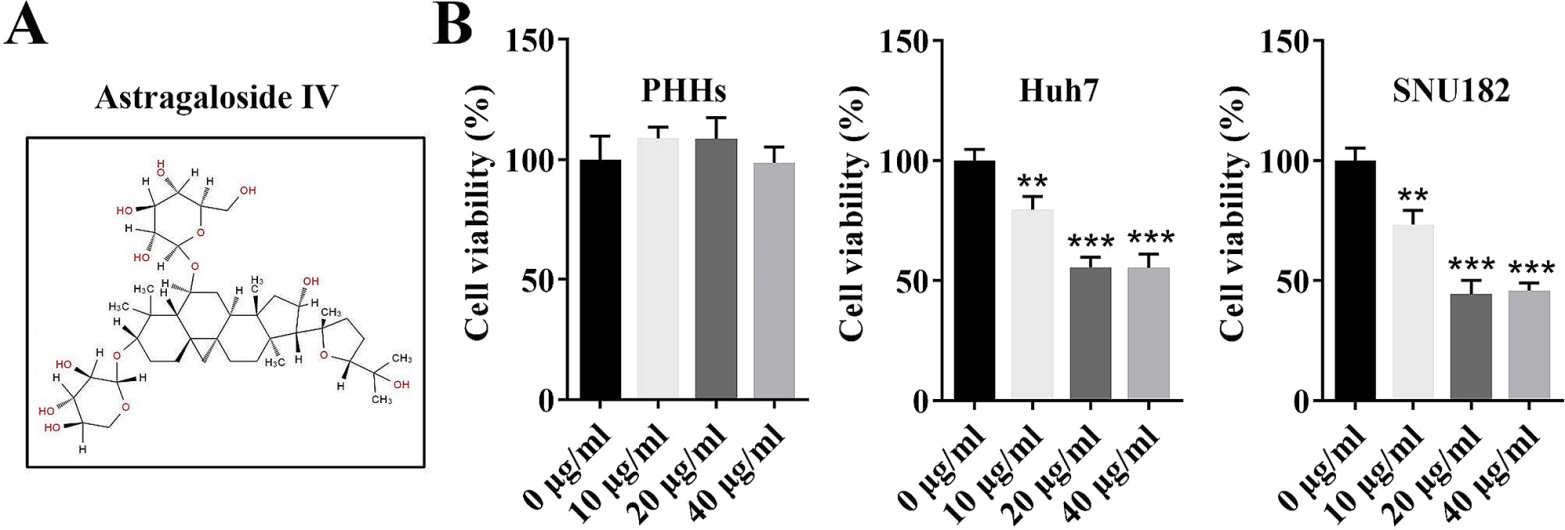
AS-IV inhibited the cell viability of SNU182 and Huh7 cell lines. (**A)** The chemical structure of AS-IV; (**B)** The cell viability of PHHs, Huh7, and SNU182 cell lines treated with different concentrations (0, 10, 20, and 40 µg/mL) of AS-IV was detected by MTT assay. ^***^P < 0.001 and ^**^P < 0.01 vs. the 0 µg/ml group. **AS-IV**, Astragaloside IV; **PHHs**, primary human hepatocytes; **MTT**, 3-(4,5)-dimethylthiahiazo (-z-y1)-3,5-di- phenytetrazoliumromide

AS-IV (MedChemExpress, Shanghai, China; C_41_H_68_O_14_, molecular weight: 784.9702; purity > 98%) was dissolved in normal saline (Beyotime, Shanghai, China) containing 0.1% dimethyl sulfoxide (DMSO, Beyotime). The PHHs, SNU-182, and Huh7 cells were treated with different doses (0, 10, 20, and 40 µg/mL) of AS-IV (Fig. 1A) for 12 h [30].

### Cell transfection

PGAM1 small hairpin (sh) RNA (sh-PGAM1), shRNA negative control (sh-NC), KAT2A overexpression vector (oe-KAT2A), and empty vector (oe-NC) used in this study were purchased from Genewiz Biotechnology Co., LTD (Suzhou, China). The SNU-182 and Huh7 cells (1 × 10^7^ cells/well) were inoculated in six-well plates (Beyotime). Transfection was performed using Lipofectamine 2000 (Yeason Biotechnology Co., LTD, Shanghai, China) according to the manufacturer’s instructions when the cell confluence reached about 80%. The cells were transfected for 48 h. After that, real-time quantitative polymerase chain reaction (RT-qPCR) was performed to detect the transfection efficiency. The knockdown sequences were as follows: sh-PGAM1, 5’-GGCACAGGTATTTGGCCTCAG-3’, and sh-NC, 5’-TTCTCCGAACGTGTCACGT-3’.

### Mutation of succinylation sites

Firstly, the GPSuc database (http://kurata14.bio.kyutech.ac.jp/GPSuc/index.php) was used to screen succinylation sites of PGAM1, and three possible PGAM1 succinylation sites, K85, K98, and K161 were chosen. Arginine mutations were introduced at K85 (K85R), K98 (K98R), and K161 (K161R) sites of PGAM1. The K85R, K98R, and K161R plasmids were designed by Genscript Biotechnology Co., LTD (Nanjing, China) and transfected into SNU182 and Huh7 cell lines for 24 h.

### 3-(4,5)-dimethylthiahiazo (-z-y1)-3,5-di- phenytetrazoliumromide (MTT) assay

MTT assay was performed using a commercial kit (Baiyue Biotechnology Co., LTD, Zhuji, China) to assess cell viability as previously described [31]. Briefly, 1 × 10^4^ cells were plated into 96-well plates and cultured at 37 °C with 5% CO_2_ for 24 h. Then, 10 µL MTT solution was added to each well. The cells were incubated at 37 °C with 5% CO_2_ for 4 h followed 100 µL formazan solution added after removing the supernatant carefully. Subsequently, the 96-well plate was placed on the oscillator (Dam industry Co., LTD, Shanghai, China) at 300 rpm/min for 10 min to dissolve the formazan completely. Finally, the OD values were measured at 570 nm by the microplate reader (Thermo Fisher).

### Western blot

The lysis buffer (Beyotime) was added to cells to extract the protein. The homogenate was homogenized in an ice bath, shaken at 4℃ for 2 h, and then centrifuged (12,000 rpm) at 4℃ for 20 min. The supernatant was then taken and stored at -80℃. The Bradford assay (Solarbio, Beijing, China) was performed to detect the protein concentration. Then, 10% SDS-PAGE (Detai Bioengineering Co. LTD., Nanjing, China) was used to separate the protein (50 µg). The protein was subsequently transferred to PVDF membrane (Detai). The membrane was blocked in 5% skim milk for 1 h, and incubated with the primary antibodies at 4 °C overnight. After that, the membrane was washed three times with Tris-buffered saline Tween (TBST, Beyotime), and the secondary antibody was incubated with the membrane at 25 °C for 1 h. Finally, protein signal detection was performed using an enhanced chemiluminescence solution (Solarbio). The used antibodies were listed as follows: succinyllysine (PTM Biolabs, Hangzhou, China; PTM-401; 1/1000), KAT2A (Abcam, Cambridge, MA, USA; ab217876; 1/1000), KAT3B (Abcam; ab275378; 1/1000), CPT1A (Abcam; ab220789; 1/1000), SIRT5 (Abcam; ab259967; 1/1000), SIRT7 (Abcam; ab259968; 1/1000), PGAM1 (Abcam; ab288376; 1/1000), glyceraldehyde 3-phosphate dehydrogenase (GAPDH) (Abcam; ab181602; 1/10,000), and goat anti-rabbit IgG (Abcam; ab205718; 1/5000).

### RT-qPCR

Firstly, TRIzol reagent (Solarbio) was used to extract total RNA from cells. Then, RNA was reverse transcribed into cDNA using the Evo M-MLV RT Kit for qPCR (Accurate Biology, Changsha, China), and the qPCR amplification experiment was performed using the SYBR Green Premix Pro Taq HS qPCR Kit (Accurate) with the reaction conditions: 95 °C for 30 s, 40 cycles of 95 °C for 5 s, 60 °C for 30 s, and a dissociation stage. The gene expression was calculated by the 2^−ΔΔCT^ method. Primers used in the present study are listed as follows: lysine acetyltransferase (KAT) 2 A, forward, 5′-GCAAGGCCAATGAAACCTGTA-3′ and reverse, 5′-TCCAAGTGGGATACGTGGTCA-3′; phosphoglycerate mutase 1 (PGAM1), forward, 5′-TTGAATACAGCGACCCAGTGG-3′ and reverse, 5′-CTATCGATGTACAGCCGAATGGTG-3′; glyceraldehyde-3-phosphate dehydrogenase (GAPDH), 5′-GACTCATGACCACAGTCCATGC-3′ and reverse, 5′-AGAGGCAGGGATGATGTTCTG-3′.

### Glucose consumption measurement

Glucose consumption in SNU182 and Huh7 cell lines was analyzed using the Screen Quest Colorimetric Glucose Uptake Assay Kit (AAT Bioquest, USA) as previously described [32]. The cells were cultured in DMEM on 96-well plates, washed with Krebs-Ringer-Phosphate-HEPES (KRPH) buffer, and incubated in 90 µL/well of Glucose Uptake Buffer for 1 h. Subsequently, cells were exposed to 10 µL/well of 2-Deoxy-D-glucose (2-DG) of 10 mM for 40 min. After that, cells were washed with KRPH buffer and lysed. Finally, 50 µL of the Uptake Assay Mixure was added to each sample and the absorbance ratio was read at 570 nm wavelength.

### Lactate production measurement

For measurement of lactate level, transfected 1 × 10^5^ SNU182 and Huh7 cell lines were seeded into six-well plates. The lactate level in the cell-cultured medium was quantified using the lactate assay kit (Abbkine Biotechnology Co., LTD, Wuhan, China) according to the manufacturer’s instructions. The sample absorbance at 530 nm was detected using the Synergy HTX Multi-Mode Microplate Reader (Agilent Technology Co. LTD., Beijing, China). The Bradford assay was used to measure protein content and the results were normalized to corresponding protein amounts.

### Measurement of extracellular acidification rate (ECAR)

The glycolytic stress test kit (Seahorse Bioscience, Agilent) was used for ECAR detection. Briefly, the cells were plated at 1 × 10^5^ cells/well in the Seahorse XF cell culture microplate. After calibration of the analyzer, sequential compound injections, including glucose, oligomycin, and 2-DG at final concentrations of 10 mM, 0.5 µM, and 50 mM were applied to test glycolytic activity. Post assay, wells were washed, cells lysed, and the Bradford assay was performed to analyze protein content. These readouts were normalized to corresponding protein amounts. The ECAR of SNU182 and Huh7 cell lines were analyzed using the Seahorse XFe24 Flux Analyzer.

### Immunofluorescence (IF) staining

IF assay was performed to observe the distribution of KAT2A and PGAM1 in SNU182 and Huh7 cell lines. The cells growing on the glass slide were soaked in phosphate buffer solution (PBS, pH 7.4, Sigma) for 3 times, and fixed by 4% paraformaldehyde (Sigma) for 15 min. Next, the sections were rinsed three times with PBS, and incubated with KAT2A and PGAM1 antibodies overnight at 4 °C, followed by incubation with the secondary antibody for 1 h at room temperature in the dark. Then, the sections were mounted with Antifade Mounting solution containing 10 mg/mL 4’6-diamidino-2-phenylindole (DAPI, Beyotime). Representative visual fields were acquired using a Leica DM5000 B microscope (Leica Microsystems, Wetzlar, Germany). The used antibodies were listed as follows: KAT2A (Abcam; ab153903; 1/500), PGAM1 (Proteintech Group, Wuhan, China; 16126-1-AP; 1/100), and goat anti-rabbit IgG (Abcam; ab150077; 1/500).

### Co-immunoprecipitation (Co-IP) assay

The interaction relationship between KAT2A and PGAM1 in SNU182 and Huh7 cell lines was detected by Co-IP assay. The cells were lysed on ice in Radio Immunoprecipitation Assay Lysis (RIPA, Sigma) buffer containing protease inhibitors for 30 min. Then, the supernatant was collected, and a small amount of it was taken as the input group. KAT2A, PGAM1, or lgG antibody (2 µg) was added into the remaining supernatant and incubated at 4 °C overnight. The protein A agarose beads (Abcam) were washed with appropriate lysis buffer (Sigma) three times. The pre-treated 10 µL protein A agarose beads were then added to the cell lysate and antibody complex and slowly shaken at 4℃ for 2 h to make the antibody conjugated with the protein A agarose beads. After the immunoprecipitation reaction, the complex was centrifuged at 4 °C, 3,000 rpm for 3 min. Next, the supernatant was discarded and the agarose beads were washed with 1 mL of lysis buffer three times. Finally, 15 µL of 2 × SDS loading buffer (Sigma) was added and boiled for 5 min. The precipitated protein was then analyzed using western blot assay. The used antibodies were listed as follows: KAT2A (Abcam; ab217876; 1/30), PGAM1 (Thermo Fisher; PA5-112233; 2 µL/mg of lysate), and goat anti-rabbit IgG (Thermo Fisher; 31,460; 1/1000).

### Protein stability assessment

Protein stability assessment was performed to verify the protein stability of PGAM1 after KAT2A overexpressing in SNU182 and Huh7 cell lines. The cells were treated with cycloheximide (CHX, 100 µg/mL, Abcam), a protein translation inhibitor, and the protein level of PGAM1 at different time points (0, 8, 16, and 24 h) was detected. The used antibodies were listed as follows: PGAM1 (Abcam; ab288376; 1/1000), GAPDH (Abcam; ab181602; 1/10,000), and goat anti-rabbit IgG (Abcam; ab205718; 1/5000).

### Animal study

A total of 24 male BALB/c (6 weeks old) mice were purchased from Charles River (Beijing, China) and housed in cages with 24℃, a 12 h alternating light/dark cycle and free access to water and food. After one-week adaptive feeding, the mice were randomly divided into four groups (*n* = 6 per group): control, AS-IV, AS-IV + Lentivirus negative control (LV-NC), and AS-IV + LV-KAT2A. Huh7 cells were infected with LV-NC and LV-KAT2A and adjusted cell density at 5 × 10^7^ cells/mL. The mice were subcutaneously injected with 100 µL LV-NC-infected or LV-KAT2A-infected Huh7 cells to establish a tumor-bearing mouse model. When the tumors reached about 50 mm^3^, AS-IV group mice were administered with AS-IV (40 mg/kg [33]) once a day via gavage. The control group mice were treated with the same amount of normal saline. Tumor volume was measured using a vernier caliper every week and quantified using the formula: Volume (mm^3^) =(length × width^2^)/2. After the fourth measurement of tumor volume, the mice were sacrificed. The tumors were isolated from all mice and weighed.

### Hematoxylin and eosin (H&E) staining

Following fixation in 4% paraformaldehyde, tumor tissues were embedded in paraffin and then transversely cut into 4 μm slices. For H&E staining, tissue sections were subjected to dewaxing with xylene, rehydration by gradient ethanol and then dyed with the H&E staining kit (Beyotime) obeying product manuals recommended by the supplier.

### Immunohistochemistry (IHC) assay

Tumor tissue paraffin Sect. (4 μm) were incubated with anti-PGAM1 (Abcam, ab279384, 1/500) at 4 °C overnight followed by incubating with the secondary antibody (Abcam, ab205719, 1/5000) at room temperature for 0.5 h. Then, the sections were stained with diaminobenzidine solution for 3 min at room temperature. After washing using moving water and sealing, the images were visualized under a microscope.

### Statistical analysis

The SPSS 21.0 software was used to analyze data. Data are expressed as mean ± standard deviation (SD). Student’s t-test was used for comparison between the two groups. One-way analysis of variance (ANOVA) was used for comparison among groups. Statistical analyses were performed using GraphPad Prism software (v8.0.1, GraphPad Software Inc., San Diego, CA, USA). *p* < 0.05 indicates that the difference is statistically significant.

## Results

### AS-IV inhibited the cell viability of SNU182 and Huh7 cell lines

In previous HCC studies, AS-IV was used at different concentrations [34, 35]. Thus, in order to choose the optimal concentration for this study, different HCC cell lines (PHHs, SNU182, and Huh7) were treated with different concentrations of AS-IV, and the cell viability was then determined by MTT assay. Results showed that AS-IV treatment showed no effects on cell viability of PHHs cell lines. Besides, the cell viability of SNU182 and Huh7 cell lines was decreased with the increasing concentration of AS-IV (0, 10, and 20 µg/mL), and 40 µg/mL could not further inhibit cell viability (Fig. 1B). Hence, AS-IV at the concentration of 20 µg/mL was chosen for further studies.

### AS-IV treatment decreased glycolysis in SNU182 and Huh7 cell lines

**Fig. 2:**
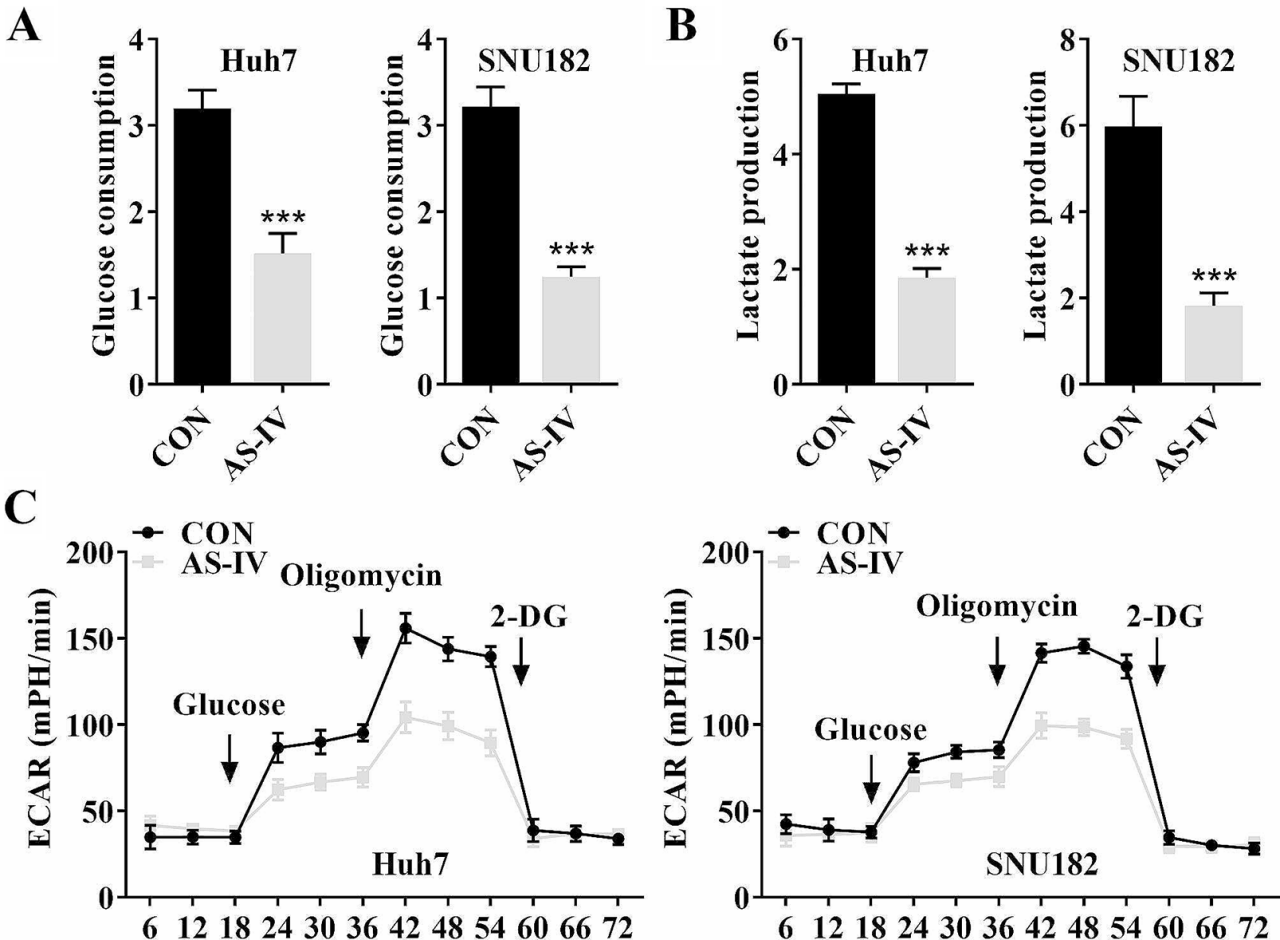
AS-IV treatment decreased glycolysis in SNU182 and Huh7 cell lines. The (**A**) glucose consumption, (**B**) lactate production, and (**C**) ECAR of SNU182 and Huh7 cell lines were detected by specific commercial kits in SNU182 and Huh7 cell lines. ***P < 0.001 vs. the CON group. **AS-IV**, Astragaloside IV; **ECAR**, extracellular acidification rate

A previous review indicates that the carcinogenic regulation of glycolysis and the multifaceted roles of glycolysis components emphasize the biological significance of tumor glycolysis [36]. Whereas, the role of AS-IV in glycolysis has only been studied in breast cancer [37]. Therefore, we focused on the glycolysis effect of AS-IV on HCC in this study. The results showed that AS-IV-treated SNU182 and Huh7 cell lines decreased glucose consumption (Fig. 2A), lactate production (Fig. 2B), and ECAR (Fig. 2C) compared with the control group.

### AS-IV treatment decreased succinylation level and KAT2A protein level in SNU182 and Huh7 cell lines

**Fig. 3:**
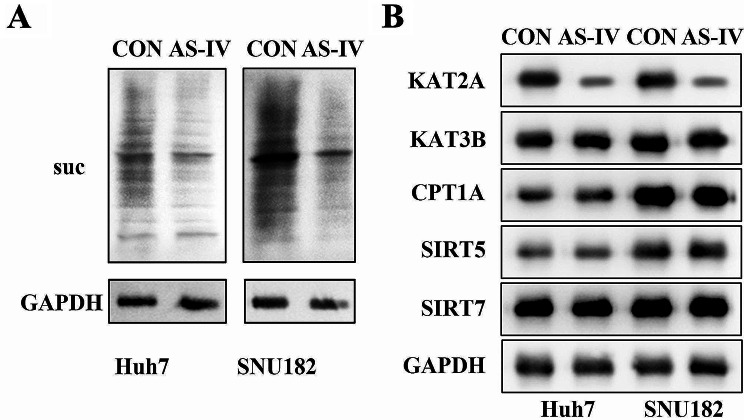
AS-IV treatment decreased succinylation level and KAT2A protein level in SNU182 and Huh7 cell lines. (**A**) The succinylation level of SNU182 and Huh7 cell lines was detected by western blot; (**B**) Western blot was used to analyze the protein levels of KAT2A, KAT3B, **CPT1A**, SIRT5, and SIRT7 in SNU182 and Huh7 cell lines. **AS-IV**, Astragaloside IV; **KAT2A**, lysine acetyltransferase 2 A; **KAT3B**, lysine acetyltransferase 3B; CPT1A, carnitine palmitoyltransferase 1 A; **SIRT**, sirtuin

Previous research elucidates the role of succinylation in the regulation of glycolysis in HCC [38]. However, the role of AS-IV in succinylation has not been found. In the present study, AS-IV-treated SNU182 and Huh7 cell lines showed downregulated succinylation level compared with the control group (Fig. 3A). Western blot was used to further explore which succinylation enzymes affected succinylation level in SNU182 and Huh7 cell lines. The results showed that the protein level of KAT2A was suppressed and that of KAT3B, CPT1A, SIRT5, and SIRT7 were not altered in AS-IV-treated SNU182 and Huh7 cell lines compared with the control group (Fig. 3B).

### Overexpression of KAT2A reversed the decreased cell viability and glycolysis in SNU182 and Huh7 cell lines caused by AS-IV treatment

**Fig. 4:**
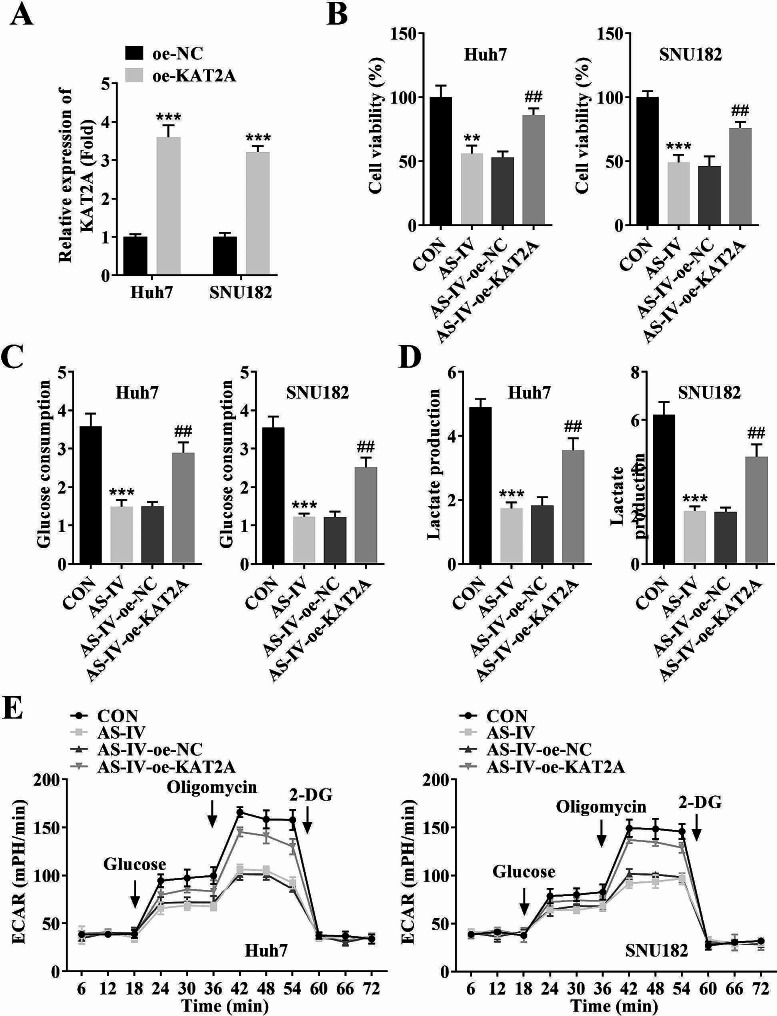
Overexpression of KAT2A reversed the decreased cell viability and glycolysis in SNU182 and Huh7 cell lines caused by AS-IV treatment. (**A**) The transfection efficiency of oe-KAT2A in SNU182 and Huh7 cell lines was detected by RT-qPCR; (**B**) The cell viability of each group was detected by MTT assay; The (**C**) glucose consumption, (**D**) lactate production, and **E**) ECAR in each group were analyzed by specific commercial kits in each group in SNU182 and Huh7 cell lines. ^***^P < 0.001 and ^**^P < 0.01 vs. the oe-NC or CON group; ^##^*P* < 0.01 vs. the AS-IV + oe-NC group. **AS-IV**, Astragaloside IV; **RT-qPCR**, reverse transcription-polymerase chain reaction; **KAT2A**, lysine acetyltransferase 2 A; **MTT**, 3-(4,5)-dimethylthiahiazo (-z-y1)-3,5-di- phenytetrazoliumromide; **ECAR**, extracellular acidification rate

To further explore the effects of KAT2A on HCC cellular processes, KAT2A overexpression plasmid was transfected into SNU182 and Huh7 cell lines, which resulted in an increased KAT2A mRNA level (Fig. 4A). Overexpression of KAT2A reversed the downregulated cell viability (Fig. 4B), glucose consumption (Fig. 4C), lactate production (Fig. 4D), and ECAR (Fig. 4E) caused by AS-IV treatment in SNU182 and Huh7 cell lines.

### PGAM1 was succinylated at K161 site

**Fig. 5:**
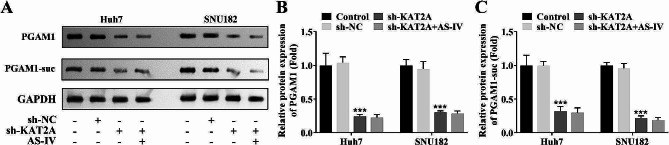
Inhibition of KAT2A downregulated the protein levels of PGAM1 and PGAM1-suc in SNU182 and Huh7 cell line. (**A**) Western blot was performed to analyze the protein levels of PGAM1 and PGAM1-suc in each group; (**B**) Quantification of Western blot results. **KAT2A**, lysine acetyltransferase 2 A; **PGAM1**, phosphoglycerate mutase 1; **suc**, succinylation

**Fig. 6:**
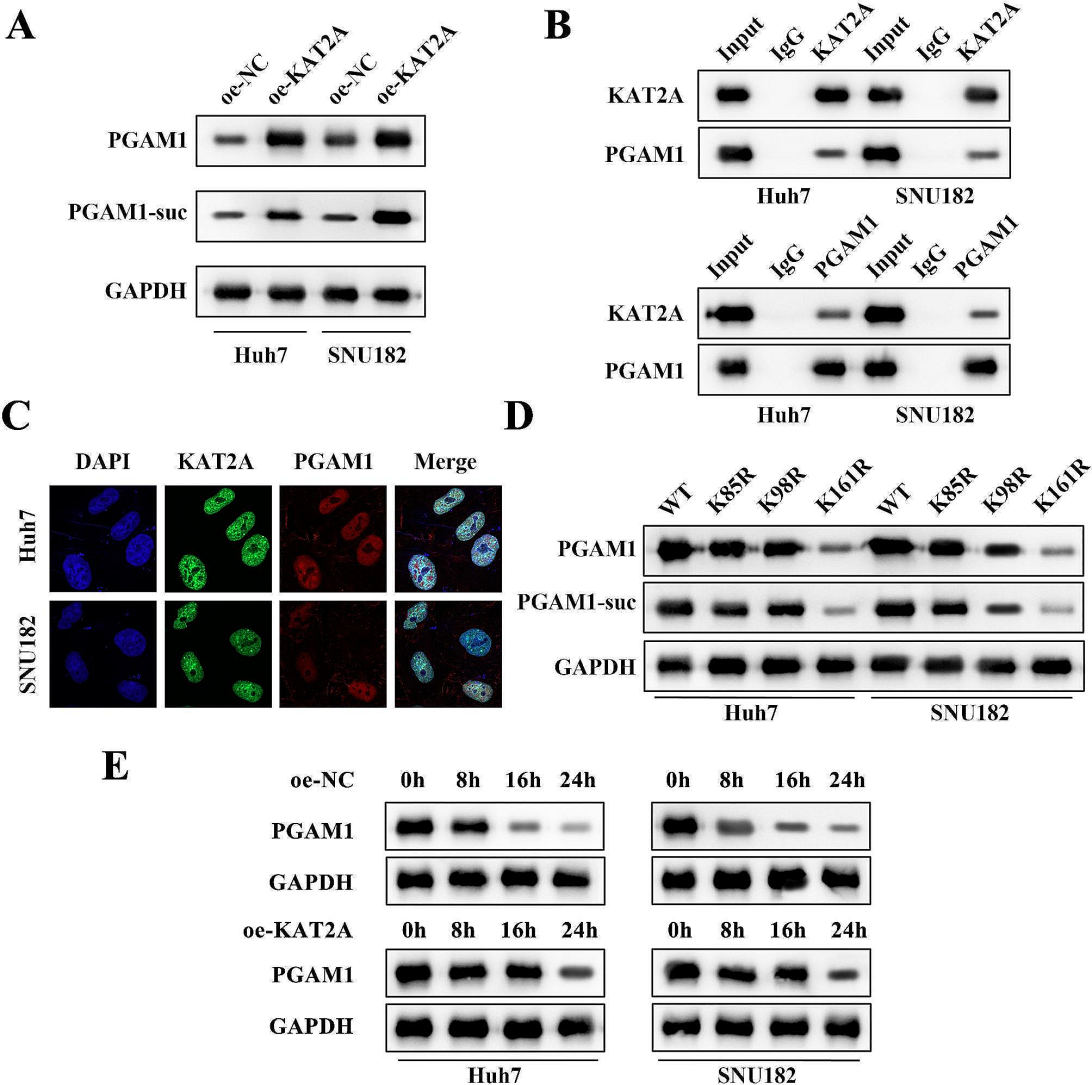
PGAM1 was succinylated at K161 site. (**A**) The protein levels of PGAM1-suc and PGAM1 in each group in SNU182 and Huh7 cell lines were analyzed by western blot; (**B**) Co-IP assay was used to analyze the interaction between KAT2A and PGAM1 in SNU182 and Huh7 cell lines; (**C**) The protein distribution of KAT2A and PGAM1 in SNU182 and Huh7 cell lines was analyzed by IF assay; (**D**) Western blot was used to determine the succinylation site of PGAM1; (**E**) The protein stability of PGAM1 was assayed by western blot at the different time points (0, 8, 16, and 24 h) after KAT2A overexpression. **KAT2A**, lysine acetyltransferase 2 A; **PGAM1**, phosphoglycerate mutase 1; **Co-IP**, co-immunoprecipitation; **IF**, immunofluorescence

In order to detect the dependence of astragaloside IV activity on KAT2A-mediated succinylation, the SNU182 and Huh7 cell lines were transfected with sh-NC, sh-KAT2A, oe-NC, and oe-KAT2A plasmids. The results indicated that inhibition of KAT2A downregulated the protein levels of PGAM1 and PGAM1-suc in SNU182 and Huh7 cell lines. Whereas, AS-IV treatment did not change the protein levels of PGAM1 and PGAM1-suc in SNU182 and Huh7 cell lines when KAT2A was knockdown (Fig. 5A-C), implying that KAT2A may lead to increased PGAM1 succinylation. Besides, KAT2A overexpression upregulated the protein levels of PGAM1 and PGAM1-suc in SNU182 and Huh7 cell lines (Fig. 6A). Co-IP assay demonstrated that KAT2A interacted with PGAM1 in SNU182 and Huh7 cell lines (Fig. 6B). IF staining suggested that KAT2A co-located with PGAM1 in SNU182 and Huh7 cell lines (Fig. 6C). The protein levels of PGAM1-suc and PGAM1 protein were decreased when the SNU182 and Huh7 cell lines were co-transfected with K161R rather than K85R and K98R, suggesting that PGAM1 was succinylated at K161 site (Fig. 6D). Protein stability assay revealed that overexpression of KAT2A enhanced the protein stability of PGAM1 in SNU182 and Huh7 cell lines compared to the control group (Fig. 6E).

### PGAM1 inhibition restored the increased cell viability and glycolysis in SNU182 and Huh7 cell lines caused by KAT2A overexpression

**Fig. 7:**
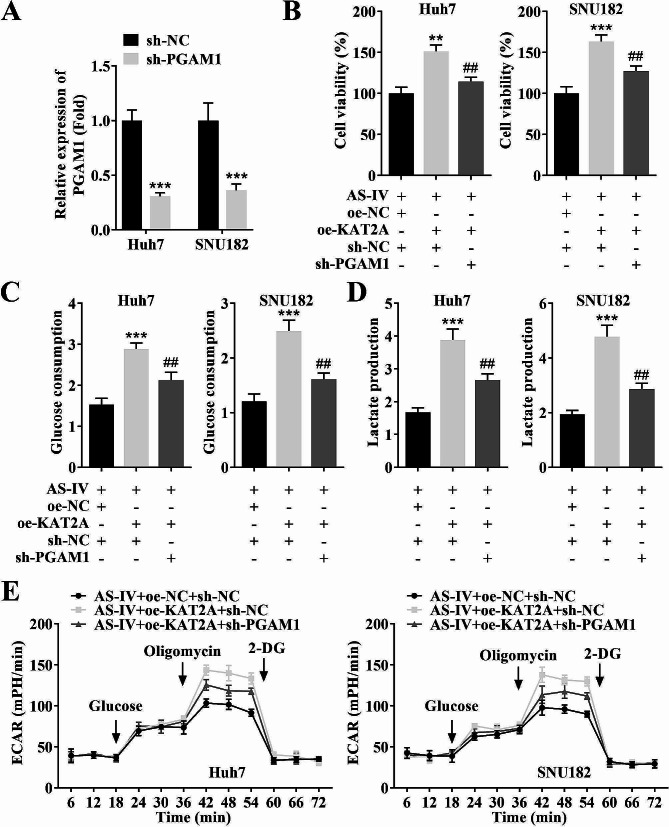
PGAM1 inhibition restored the increased cell viability and glycolysis in SNU182 and Huh7 cell lines caused by KAT2A overexpression. (**A**) The transfection efficiency of sh-KAT2A in SNU182 and Huh7 cell lines was detected by RT-qPCR; (**B**) The cell viability of each group was detected by MTT assay; The (**C**) glucose consumption, (**D**) lactate production, and (**E**) ECAR in each group were analyzed by specific commercial kits in SNU182 and Huh7 cell lines. ^***^P < 0.001 and ^**^P < 0.01 vs. the sh-NC or AS-IV + oe-NC + sh-NC group; ^##^*P* < 0.01 vs. the AS-IV + oe-KAT2A + sh-NC group. **AS-IV**, Astragaloside IV; **RT-qPCR**, reverse transcription-polymerase chain reaction; **KAT2A**, lysine acetyltransferase 2 A; **MTT**, 3-(4,5)-dimethylthiahiazo (-z-y1)-3,5-di- phenytetrazoliumromide; **ECAR**, extracellular acidification rate

To further explore the effects of KAT2A and PGAM1 on glycolysis in HCC, KAT2A overexpression and PGAM1 knockdown plasmids were transfected into SNU182 and Huh7 cell lines. The PGAM1 expression was downregulated after sh-PGAM1 transfection (Fig. 7A). PGAM1 inhibition restored the increased cell viability (Fig. 7B), glucose consumption (Fig. 7C), lactate production (Fig. 7D), and ECAR (Fig. 7E) caused by KAT2A overexpression in SNU182 and Huh7 cell lines.

### AS-IV suppressed tumor growth though inhibiting KAT2A-mediated succinylation of PGAM1

**Fig. 8:**
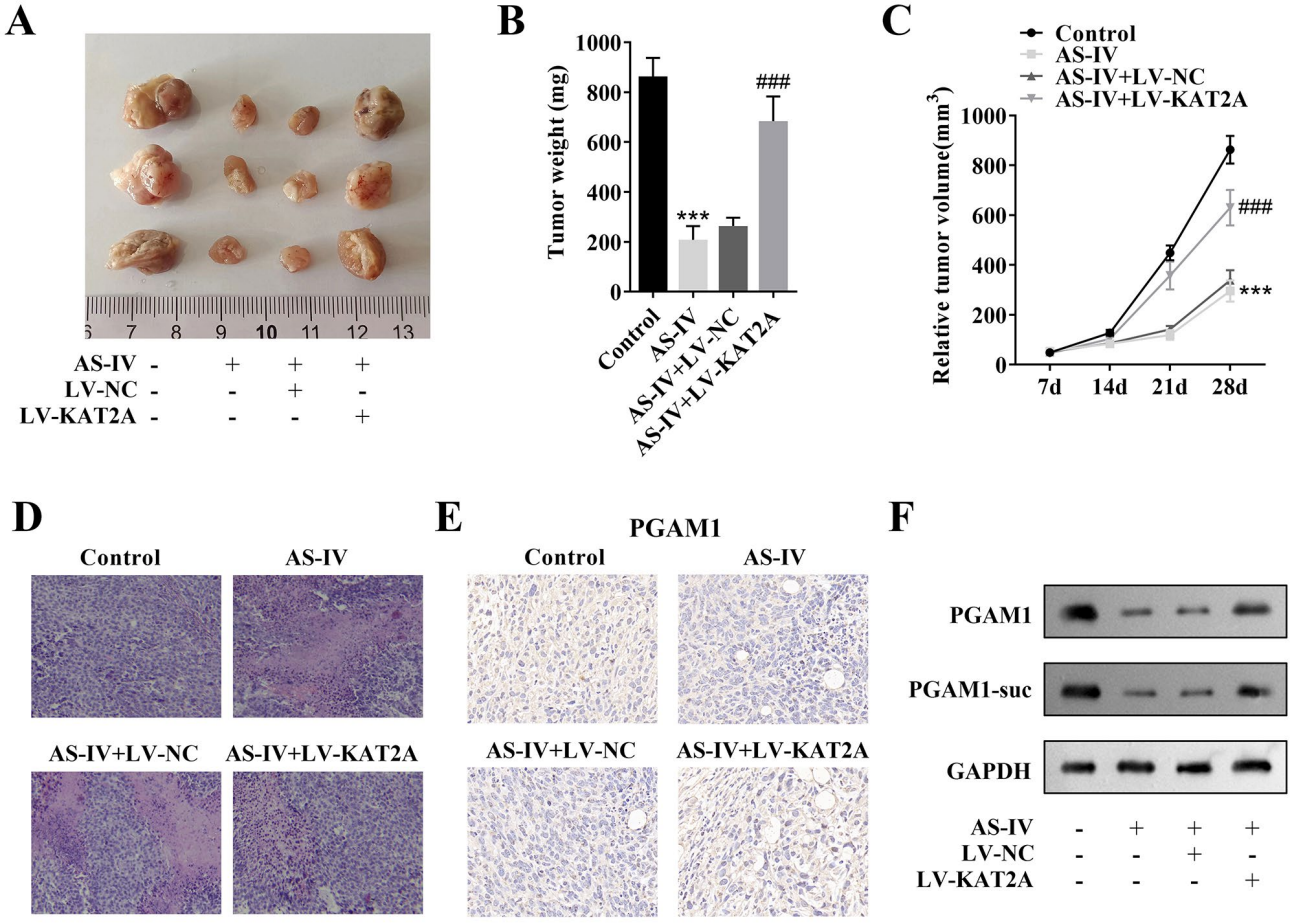
AS-IV suppressed tumor growth though inhibiting KAT2A-mediated succinylation of PGAM1. (**A**) The represent images of tumors from mice of each group; (**B**) Tumor weight of each group; (**C**) Tumor volume was measured weekly; (**D**) Histological examination of the morphological changes in tumors from mice of each group; (**E**) IHC assessed PGAM1 protein level in tumors; (**F**) Western blot was performed to detect the protein levels of PGAM1 and PGAM1-suc in each group. ^***^P < 0.001 vs. the control group; ^###^*P* < 0.01 vs. the AS-IV + LV-NC group. **AS-IV**, Astragaloside IV; **KAT2A**, lysine acetyltransferase 2 A; **PGAM1**, phosphoglycerate mutase 1; **IHC**, immunohistochemical; **succ**, succinylation

Finally, we established the tumor-bearing mouse model to explore the role of AS-IV and KAT2A in vivo. The results showed that AS-IV reduced tumor size and weight compared with the control group, while KAT2A overexpression reversed the effects induced by AS-IV (Fig. 8A-C). H&E results showed that tumor cells in the control group were plump and intact, with large nuclei. After treatment with AS-IV, the tumor cells exhibited a large necrotic region and were stained darker, while the results were reversed after KAT2A overexpression (Fig. 8D). The protein levels of PGAM1 and PGAM1-suc were downregulated by AS-IV. Moreover, the levels PGAM1 and PGAM1-suc were increased by KAT2A overexpression, compared with the AS-IV + vector group (Fig. 8E and F). Taken together, AS-IV suppressed tumor growth via inhibiting KAT2A-mediated succinylation of PGAM1.

## Discussion

With the acceleration of TCM modernization, increasing evidence shows that many TCM components have significant anticancer activity [14]. In the present study, it was found that the cell viability was gradually decreased with an increasing AS-IV concentration (0, 10, and 20 µg/mL) in HCC cells. Based on these results, 20 µg/mL AS-IV concentration was considered optimal and hence selected for further experiments. In addition, AS-IV treatment downregulated the glycolysis in HCC cells, suggesting that AS-IV might play a suppressive role in the HCC progression. Similarly, studies have reported that the proliferation, migration, and invasion of HCC and lung cancer cells following AS-IV treatment were substantially attenuated [6, 13]. However, the effects of AS-IV on glycolysis have not been researched. Nonetheless, Bu-Shen-Jian-Pi-Fang, a long-standing TCM, is involved in multiple targets and signaling pathways related to tumorigenesis and glycolysis metabolism in clear cell renal cell carcinoma [39]. Additionally, shikonin, a natural naphthoquinone compound, was also reported to inhibit rheumatoid arthritis by regulating glycolysis [40].

It has been reported that the alteration of glycolysis was associated with succinylation [41], thus, in this study, we examined the levels of succinylation level in SNU182 and Huh7 cell lines. The results indicated that the succinylation was decreased after AS-IV treatment, implying that the progression of HCC might be related to succinylation. Moreover, overexpression of KAT2A reversed the decreased cell viability and glycolysis caused by AS-IV treatment, indicating that KAT2A-mediated succinylation might be oncogenic. Similarly, high expression of KAT2A promotes the proliferation of nasopharyngeal carcinoma cells [42]. Additionally, KAT2A has been found to promote hepatitis B virus transcription and replication through succinylation modification [43]. A previous study demonstrated that PGAM1 is a histone acetyltransferase 1 downstream regulatory gene, another succinyltransferase, involved in various tumors progression [44]. Thus, we examined the relationship between KAT2A and PGAM1 in this study, and the results showed that overexpression of KAT2A upregulated the protein levels of PGAM1 and PGAM1-suc. Moreover, KAT2A interacted with PGAM1 in HCC cells, suggesting that PGAM1 was a downstream regulatory target for KAT2A in HCC. Additionally, it was found that silencing of PGAM1 rescued the increased cell viability and glycolysis caused by KAT2A overexpression in AS-IV-treated HCC cell lines, further supporting the above results. KAT2A is often regarded as an acetyltransferase, and the succinylation of KAT2A has been poorly studied. A study demonstrates that KAT2A could act as a histone succinyltransferase from succinyl-CoA directly to histone H3 lysine 79 (H3K79) from a molecular perspective, which is important for the regulation of gene expression in tumor cells [45]. The specific mechanism of KAT2A in succinylation in tumors remains to be further verified. Additionally, our results found that PGAM1 was succinylated at K161 site, which has not been reported before. This could provide a reference for further study to explore the specific mechanism of PGAM1 in HCC or other cancers. In tumor-bearing mice, AS-IV treatment suppressed the tumor growth and the succinylation of PGAM1, while KAT2A overexpression reversed these results. These results suggested that AS-IV suppressed tumor growth though inhibiting KAT2A-mediated succinylation of PGAM1.

There are several limitations in this study. Firstly, we only used SNU-182 and HuH-7 cell lines for analysis, and it is necessary to investigate more HCC cell lines in future studies. The underlying mechanism of AS-IV on HCC may be complex, including but not limited to PGAM1 succinylation. Moreover, it is ideal to detect the effects of KAT2A-overexpression and knockdown in the presence of succinyl-resistant and succinyl-like mutants of K161. In addition, we have to admit that the resolution of the microscopy we used in the IF study is low. We will further address these deficiencies as experimental conditions permit in the future.

As shown in supplemental Fig. S1, our study first found the inhibitory effects of AS-IV on HCC cell viability and glycolysis. Subsequent studies suggested that the above inhibitory effects were achieved by downregulating KAT2A. Further mechanistic studies indicated that KAT2A deficiency inhibited PGAM1 and its succinylation expression, thus suppressing the cell viability and glycolysis. In conclusion, AS-IV inhibited cell viability and glycolysis of HCC by regulating KAT2A-mediated succinylation of PGAM1, which might provide new insights into the treatment of in HCC.

### Electronic supplementary material

Below is the link to the electronic supplementary material.

Supplementary Material 1

## Data Availability

The datasets used and/or analysed during the current study are available from the corresponding author on reasonable request.
