# Supplementary figures and images for: Astragaloside IV inhibits cell viability and glycolysis of hepatocellular carcinoma by regulating KAT2A-mediated succinylation of PGAM1
Source: BMC Cancer. 2024 Jun 4;24:682. doi: 10.1186/s12885-024-12438-9 (PMC11151566; doi:10.1186/s12885-024-12438-9)

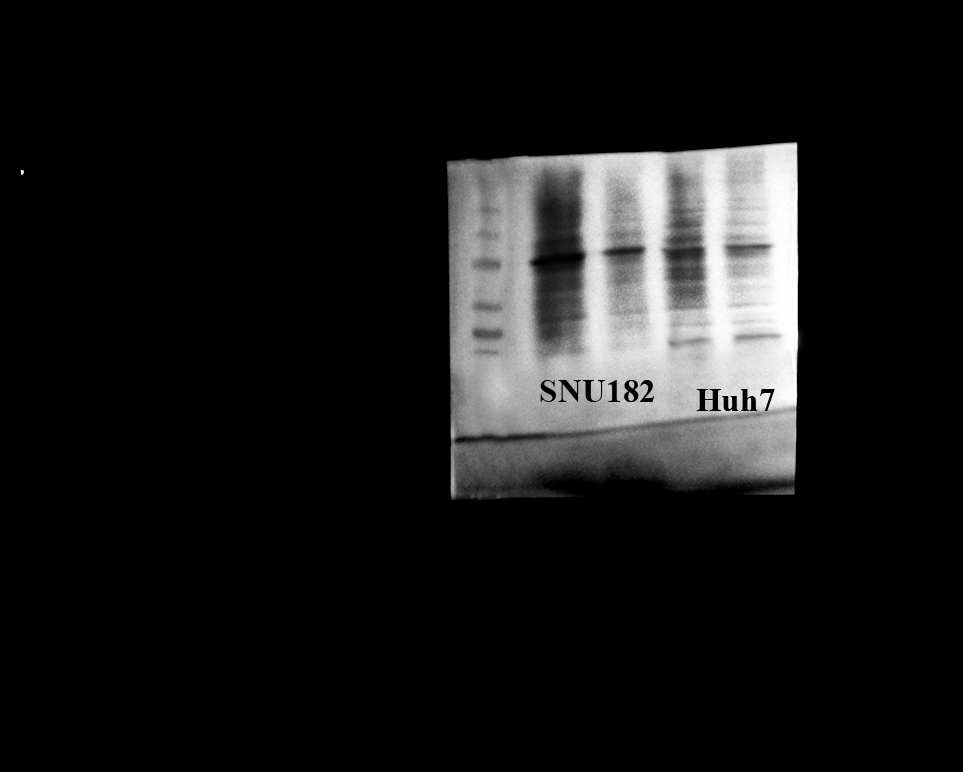

Supplement: Supplementary file 1 — Supplementary Material 1 [file 12885_2024_12438_MOESM1_ESM.png]
